# Supplementary material for: Modeling the Worldwide Spread of Pandemic Influenza: Baseline Case and Containment Interventions
Source: PLoS Med. 2007 Jan 23;4(1):e13. doi: 10.1371/journal.pmed.0040013 (PMC1779816; doi:10.1371/journal.pmed.0040013)
Supplement: Supplementary file 1 [file pmed.0040013.sd001.pdf]

Modeling the world-wide spread of pandemic influenza: baseline case and containment interventions
Supporting information

V. Colizza[1,2], A. Barrat[2,3], M. Barthélemy[1,4], A.-J. Valleron[5,6] and A. Vespignani[1,2]

November 7, 2006

[1] School of Informatics and Biocomplexity Center, Indiana University, Bloomington, 47406, IN, USA

[2] Complex Networks Lagrange Laboratory (CNLL), ISI Foundation, Turin, Italy

[3] Unité Mixte de Recherche du CNRS UMR 8627, Bâtiment 210, Université de Paris-Sud, F-91405 Orsay, France

[4] CEA-DIF, Centre d'Etudes de Bruyères-Le-Chatel, BP12, F-91680, France,

[5] Unité de Recherche "Epidémiologie, Systèmes d'Information et Modélisation", INSERM (U707), Université Pierre et Marie Curie, 27, rue Chaligny, Paris, F-75012, France

[6] Hopital Saint-Antoine, Assistance Publique-Hopitaux de Paris, Paris, France

Global stochastic epidemic model

We consider a global stochastic epidemic model [1] which takes into account the complete International Air Transport Association (IATA) [2] database, composed of 3100 airports and 17182 weighted edges accounting for the traffic of passengers between pairs of airports. Inside each

city we assume a standard compartmentalization of the population in which every individual belongs to a certain compartment; the compartments used allow for a detailed description of a typical evolution of the actual disease, by including the incubation period and the asymptomatic course of influenza [7, 8]. Individuals in each city can change compartment by following the infection dynamics and are also allowed to travel from one city to another by following the routes of the airline transportation network.

Details of the compartmentalization, in the baseline case and in the intervention scenario, are shown in the diagrams of Figure 2 of the manuscript.

In each city j the population is given by $N_j = S_j(t) + L_j + I_j^t(t) + I_j^{nt}(t) + I_j^a(t) + R_j(t)$, where $S_j(t)$, $L_j(t)$, $I_j^t(t)$, $I_j^{nt}(t)$, $I_j^a(t)$ and $R_j(t)$ represent at time t the number of susceptible, latent, symptomatic traveling, symptomatic not traveling, asymptomatic infected and recovered individuals, respectively. The model in the baseline scenario is described by the following set of discretized epidemic Langevin equations:

$$
\begin{aligned}
S_j(t+\Delta t) - S_j(t) &= -\beta \frac{(I_j^t + I_j^{nt} + r_\beta I_j^a) S_j}{N_j} \Delta t + \sqrt{\beta \frac{I_j^t S_j}{N_j} \Delta t}\, \eta_{\beta_t,j}(t) + \\
&\quad + \sqrt{\beta \frac{I_j^{nt} S_j}{N_j} \Delta t}\, \eta_{\beta_{nt},j}(t) + \sqrt{r_\beta \beta \frac{I_j^a S_j}{N_j} \Delta t}\, \eta_{r_\beta,j}(t) + \Omega_j(\{S\}) \qquad \text{(S1)} \\
L_j(t+\Delta t) - L_j(t) &= \beta \frac{(I_j^t + I_j^{nt} + r_\beta I_j^a) S_j}{N_j} \Delta t - \epsilon L_j \Delta t - \sqrt{\beta \frac{I_j^t S_j}{N_j} \Delta t}\, \eta_{\beta_t,j}(t) + \\
&\quad - \sqrt{\beta \frac{I_j^{nt} S_j}{N_j} \Delta t}\, \eta_{\beta_{nt},j}(t) - \sqrt{r_\beta \beta \frac{I_j^a S_j}{N_j} \Delta t}\, \eta_{r_\beta,j}(t) + \\
&\quad + \sqrt{\epsilon L_j \Delta t}\, \eta_{\epsilon,j}(t) + \Omega_j(\{L\}) \qquad \text{(S2)} \\
I_j^t(t+\Delta t) - I_j^t(t) &= p_t(1-p_a)\epsilon L_j \Delta t - \mu I_j^t \Delta t - p_t(1-p_a)\sqrt{\epsilon L_j \Delta t}\, \eta_{\epsilon,j}(t) + \\
&\quad + \sqrt{\mu I_j^t \Delta t}\, \eta_{\mu_t,j}(t) + \Omega_j(\{I^t\}) \qquad \text{(S3)} \\
I_j^{nt}(t+\Delta t) - I_j^{nt}(t) &= (1-p_t)(1-p_a)\epsilon L_j \Delta t - \mu I_j^{nt} \Delta t + \\
&\quad -(1-p_t)(1-p_a)\sqrt{\epsilon L_j \Delta t}\, \eta_{\epsilon,j}(t) + \sqrt{\mu I_j^{nt} \Delta t}\, \eta_{\mu_{nt},j}(t) \qquad \text{(S4)} \\
I_j^a(t+\Delta t) - I_j^a(t) &= p_a \epsilon L_j \Delta t - \mu I_j^a \Delta t - p_a \sqrt{\epsilon L_j \Delta t}\, \eta_{\epsilon,j}(t) +
\end{aligned}
$$

$$
\begin{aligned}
& +\sqrt{\mu I_j^a \Delta t}\, \eta_{\mu_a,j}(t) + \Omega_j(\{I^a\}) && \text{(S5)} \\
R_j(t+\Delta t) - R_j(t) \;=\; & \mu(I_j^t + I_j^{nt} + I_j^a)\Delta t - \sqrt{\mu I_j^t \Delta t}\, \eta_{\mu_t,j}(t) - \sqrt{\mu I_j^{nt} \Delta t}\, \eta_{\mu_{nt},j}(t) + \\
& -\sqrt{\mu I_j^a \Delta t}\, \eta_{\mu_a,j}(t) + \Omega_j(\{R\}), && \text{(S6)}
\end{aligned}
$$

where $\eta_{\beta_t,j}$, $\eta_{\beta_{nt},j}$, $\eta_{r_\beta,j}$, $\eta_{\epsilon,j}$, $\eta_{\mu_t,j}$, $\eta_{\mu_{nt},j}$, $\eta_{\mu_a,j}$ are statistically independent Gaussian random variables with zero mean and unit variance and Δt represents the time step set to 1 day.

The set of Langevin equations are coupled by the stochastic transport operator Ω_j which describes the movements of individuals traveling from one city to another. Following ref.[1], the transport term is a function of the air traffic flows between two connected cities and represents the net balance of passengers who left and entered city j.

The number of passengers of category X traveling from a city j to a city ℓ is an integer random variable, in that each of the X_j potential travellers has a probability $p_{j\ell} = w_{j\ell}/N_j$ to go from j to ℓ. In each city j the number of passengers $\xi_{j\ell}$ traveling on each connection $j \to \ell$ at time t define a set of stochastic variables which follows the multinomial distribution

$$
P(\{\xi_{j\ell}\}) = \frac{X_j!}{(X_j - \sum_\ell \xi_{j\ell})! \prod_\ell \xi_{j\ell}!} (1 - \sum_\ell p_{j\ell})^{(X_j - \sum_\ell \xi_{j\ell})} \prod_\ell p_{j\ell}^{\xi_{j\ell}}, \qquad \text{(S7)}
$$

where $(1 - \sum_\ell p_{j\ell})$ is the probability of not traveling, and $(X_j - \sum_\ell \xi_{j\ell})$ identifies the number of non traveling individuals of category X. We use standard numerical subroutines to generate random numbers of travellers following these distributions.

The transport operator in each city j is therefore written as

$$
\Omega_j(\{X\}) = \sum_\ell (\xi_{\ell j}(X_\ell) - \xi_{j\ell}(X_j)), \qquad \text{(S8)}
$$

where the mean and variance of the stochastic variables are $\langle \xi_{j\ell}(X_j) \rangle = p_{j\ell} X_j$ and $\mathrm{Var}(\xi_{j\ell}(X_j)) = p_{j\ell}(1 - p_{j\ell}) X_j$. Direct flights as well as connecting flights up to two-legs flights can be considered.

In addition to the previous fluctuations, the transport operator is in general affected by fluctuations coming from the fact that the occupancy rate of the airplanes is not 100%. To take into account such fluctuations, we assume that on each connection (j, ℓ) the flux of passengers at

time t is given by a stochastic variable

$$\tilde{w}_{j\ell} = w_{j\ell}(\alpha + \eta(1-\alpha)) \tag{S9}$$

where $\alpha = 0.7$ corresponds to the average occupancy rate of order of 70% provided by IATA and η is a random number drawn uniformly in the interval $[-1, 1]$ at each time step.

This system of differential equations can be numerically integrated to obtain the evolution of the infection dynamics inside each city. A major issue in the integration of the epidemic equations is the consideration of the discrete nature of the individuals and the preservation of the symmetry of the noise terms. For this reason we adopt an integration procedure that separates each variable X_j into its integer part $[X_j]$ and its non-integer remaining part $\tilde{X}_j$ and we write the corresponding time evolution for these variables [19, 1]. This procedure is implemented at all time steps in each urban area j and provides a discrete output which preserves the correct integration of the equations. In addition, it is worth noticing that the derivation of the Langevin equations from the master equations describing the evolution of the probabilities associated to the infection dynamics is valid under the assumption of large population in each compartment for each city [18, 16, 17]. Therefore for the sake of mathematical rigor, in each compartment with less than 10^3 individuals we describe the evolution of the disease by fully considering discrete binomial or multinomial processes. This condition is checked at all time steps for all compartments in all urban areas. In particular two different kind of processes in the infection dynamics have to be considered: the generation of new cases (i.e. the generation of latents through the contact of susceptible and infectious individuals) and the transition of individuals from one compartment to another (e.g. from latent to infectious, from infectious to recovered). For the first class of process it is assumed that each susceptible will become in contact with an infectious individual with probability $\beta I_j(t)/N_j$, so that the number of new infections in city j is extracted from a binomial distribution with probability $\beta I_j(t)/N_j$ and number of trials $S_j(t)$. Analogously, the number of individuals changing compartment in a transition process (e.g. in city j: $L \to I$ with rate ϵ) is extracted from a binomial distribution with probability given by the rate of transition (in the previous example, ϵ) and number of trials given by the number of

individuals in the compartment at time t (in the previous example, $L_j(t)$).

In case of multiple generation of new cases (due e.g. to susceptible individuals coming into contact with asymptomatic and symptomatic infectious individuals) and transition processes from a single compartment to more than one (e.g. latent becoming either symptomatic or asymptomatic), the binomial is substituted by a multinomial distribution, whose parameters are given by the probabilities associated to the single processes considered and the population of the given compartment. As an example, the number of latent becoming either asymptomatic or symptomatic traveling/non-traveling is extracted from a multinomial distribution based on the probabilities $p_{L\to I^a} = p_a\epsilon$, $p_{L\to I^t} = (1-p_a)p_t\epsilon$ and $p_{L\to I^{nt}} = (1-p_a)(1-p_t)\epsilon$, respectively, out of the total pool of latent individuals at time t, $L(t)$.

When the assumption of large populations is satisfied, the epidemic evolution is then obtained through the integration of the Langevin equations, as described above. Results are in agreement with those obtained from a fully discretized version of the model in which all processes of the infection dynamics are described through multinomial distributions.

Infectious period under AV treatment

The efficacy of the AV treatment is modeled through a reduction of the infectiousness of treated individuals and a reduction of the total infectious period [7, 8, 10, 9, 20, 22]. Since the compartmentalization considered does not allow to track the time since infection occurred for each individual, we consider a reduction of x days of the *average* infectious period. We assume that infectious individuals are allowed to recover before eventually being detected and administered an AV dose. Therefore, under AV treatment the transition from infectious individual to infectious under treatment – $I^{t,nt}$ to I_{AV} – is assumed to occur with rate $(1-\mu)p_{AV}$, while the rate of transition directly to the recovered compartment is left unchanged. The average period μ_{AV}^{-1} spent in the I_{AV} compartment is then obtained by reducing the average total infectious time by

$x\Delta t$:

$$\frac{1}{\mu + (1-\mu)p_{AV}} + \frac{1}{\mu_{AV}} = \frac{1}{\mu} - x\,, \qquad \text{(S10)}$$

where the first term in the l.h.s. of the equation represents the average time spent by a treated individual in the $I^{t,nt}$ compartment. From the equation above we obtain the following expression for the average time spent in the treated compartment:

$$\mu_{AV}^{-1} = \mu^{-1} - x - \frac{1}{\mu + (1-\mu)p_{AV}}\,, \qquad \text{(S11)}$$

which clearly depends on the distribution rate p_{AV}. Indeed, a large value of p_{AV} refers to a prompt identification of cases and subsequent drug administration. Plugging into eq. S11 the values of the parameters considered (μ), we obtain different values of the rate of transition μ_{AV} for different reductions x and rate distributions p_{AV}.

Some values of x and p_{AV} lead to a value of μ_{AV}^{-1} smaller than 1. Since our time scale is $\Delta t = 1$ day, in those cases we fix μ_{AV}^{-1} to its smallest possible value, i.e. $\mu_{AV}^{-1} = 1$, and reduce the infectiousness $\beta(1 - AVE_I)$ of the treated individuals by an appropriate scaling factor s_β in order to effectively compensate the longer average period spent under treatment, imposed by time scale constraints. In this way, it is also possible to study reductions x smaller than 1 day (see in the following the sensitivity analysis on the reduction of the infectious period under treatment).

Estimate of the reproductive ratio with AV treatment

While it is possible to give an analytic expression of the reproductive rate for the compartmentalization adopted in the baseline case as provided in the manuscript, the same task becomes more difficult when AV intervention is considered because of the time delays due to the interplay of travel and AV use. In the following we provide a rough estimate of the reproductive rate under the assumption that AV intervention is put in place since the start of the pandemic (quite

unrealistic), with no additional delays. In this case, the effective $R_{0,AV}$ would be:

$$\begin{aligned} R_{0,AV} &= \frac{\beta}{\mu}\Big\{ r_\beta p_a \; + \; (1-p_a)\frac{\mu}{\mu+(1-\mu)p_{AV}} \; + \\ &+ (1-p_a)\frac{(1-\mu)p_{AV}}{\mu+(1-\mu)p_{AV}}\left[\frac{\mu}{\mu+(1-\mu)p_{AV}} + s_\beta(1-AVE_I)\frac{\mu}{\mu_{AV}}\right]\Big\} \end{aligned} \tag{S12}$$

where the r.h.s. of the equation represents the contributions of *(i)* asymptomatic individuals, *(ii)* symptomatic non-treated individuals and *(iii)* symptomatic treated individuals, respectively. In order to have a quantitative estimate of the value of $R_{0,AV}$ with respect to the reproductive rate R_0 in absence of AV, we can compute the ratio:

$$\frac{R_{0,AV}}{R_0} = \frac{r_\beta p_a + (1-p_a)\Big\{\frac{\mu}{\mu+(1-\mu)p_{AV}} + \frac{(1-\mu)p_{AV}}{\mu+(1-\mu)p_{AV}}\left[\frac{\mu}{\mu+(1-\mu)p_{AV}} + s_\beta(1-AVE_I)\frac{\mu}{\mu_{AV}}\right]\Big\}}{r_\beta p_a + (1-p_a)} . \tag{S13}$$

For a distribution rate of $p_{AV} = 0.7/day$ and assuming a reduction of the infectious period $x = 1$ day, the value of the ratio is $R_{0,AV}/R_0 \simeq 0.77$, so that for $R_0 = 1.5$ the estimate for the effective $R_{0,AV}$ gives a value $\simeq 1.15$, i.e. slightly larger than 1. However, this estimation does not take into account the fact that at initial stages of the original outbreak and of all secondary outbreaks the reproductive rate is not lowered by the use of AV treatment since the intervention takes place with a characteristic delay. The interplay of the travel and of the delays with which intervention strategies are put in place allow therefore the virus to spread out of the source, even in case of a high distribution rate of AV doses – $p_{AV} = 0.7$ – and reproductive rates $R_0 \simeq 1.5$.

Initial conditions and geographical subdivision

At the beginning of the simulation, the entire population is considered to be susceptible. The population vulnerability to an H5N1-like pandemic is indeed expected to be universal, since the human immune system will have no pre-existing immunity to a new virus [11]. We simulate the start of the pandemic influenza on several different initial dates, in order to assess the impact of seasonal patterns on the global spread. Numerical simulations start with a single symptomatic

infected individual in a given initially infected city. A sensitivity analysis of the model with respect to initial conditions has been performed by specifying different origins of the pandemic outbreak, in order to assess the impact of seasonality and traffic flows on the spatio-temporal pattern of the global spread. Rural cities as well as big metropolitan areas have been considered as the initially infected center.

Though the integration procedure of the equations reported in the previous section allows for a detailed description of the pandemic evolution at the city level, we focused on 6 different regions of the world in order to have a global view of what could happen in different zones and hemispheres. We considered the following 6 regions: North America, Western Europe, Central America, South East Asia, Lower South America, Oceania. Table S1 reports the list of countries belonging to each region considered.

N America	W Europe		C America		SE Asia	LS America	Oceania
United States	Denmark	Belgium	Mexico	Colombia	Indonesia	Argentina	Australia
Canada	United Kingdom	Greece	Honduras	Peru	Myanmar	Chile	New Zealand
	Spain	Portugal	Belize	Venezuela	Malaysia	Uruguay	
	Switzerland	Ireland	Guatemala	French Guyana	Philippines		
	Norway	Gibraltar	Costa Rica	Bolivia	Thailand		
	Iceland	Austria	Nicaragua	Ecuador	Cambodia		
	Germany	Malta	Panama	Suriname	Vietnam		
	France	Finland	El Salvador	Brazil	Singapore		
	Sweden	Luxembourg			Lao People's DR		
	Italy	Monaco			Hong Kong		
	Netherlands						

Table S1: List of countries included in each region considered. Three different colors are used to represent the three different climate zones each region belongs to; from left to right: Northern hemisphere, Tropical climate zone, Southern hemisphere.

All other countries included in the model are listed hereafter in no particular order: French Polynesia, Egypt, Algeria, South Africa, United Arab Emirates, Russian Federation, China, Yemen, Iran, Cote D'Ivoire, Ethiopia, Saud Arabia, Nigeria, Ghana, Turkey, Jordan, Mauritania, Solomon Islands Morocco, Japan, Papua New Guinea, India, Paraguay, Marshall Islands, Cook Islands, Niger, Libya, Kazakhstan, Syrian Arab Rep., Madagascar, Antigua & Barbuda,

Mozambique, Western Samoa, Tanzania, Romania, Erythrea, Turkmenistan, Bahamas, Kenya, Aruba, Vanuatu, Cuba, Mongolia, Anguilla, Uzbekistan, Bahrain, Azerbaijan, Botswana, Somalia, Bermuda, Nepal, Yugoslavia, Lebanon, Cameroon, Central African Rep., Barbados, Pakistan, Gambia, Burundi, Mali, Malawi, Gabon, New Caledonia, Bosnia-Herzegovina, Democratic Rep. of Congo, Bulgaria, Neth. Antilles, Burkina Faso, Puerto Rico, St. Vincent Grenadine, Czech Rep., Dominican Rep., Equatorial Guinea, Slovakia, Hungary, Zimbabwe, Georgia, Cape Verde Islands, Croatia, Brunei, Poland, Bangladesh, Congo, Angola, Haiti, French Guyana, Republic of Korea, Guinea, Sri Lanka, Taiwan, Greenland, Benin, Grenada, Senegal, Ukraine, Cayman Islands, Dominica, Tunisia, Sudan, Qatar, Guadeloupe, Tajikistan, Mayotte, Uganda, Cyprus, British Virgin Islands, Namibia, Israel, Tonga, Armenia, Martinique, Sierra Leone, Korea Dem. People's Rep., Kyrgyzstan, St. Pierre & Miquelon, Tuvalu, Wallis & Futuna Is., Maldives, Turks & Caicos Is., Guyana, Gibraltar, Guam, Comoros, Fiji, Nauru, Djibouti, Johnston Island, Zambia, Afghanistan, Rwanda, Oman, Jamaica, Moldova, Caroline Is., Lithuania, Kuwait, Togo, Slovenia, Macau, Belarus, Liberia, Mauritius, Lesotho, Swaziland, Chad, St. Kitts & Nevis, Norfolk Island, American Samoa, Macedonia, Guinea Bissau, Bhutan, Sao Tome & Principe, Trinidad & Tobago, Seychelles, Latvia, Northern Mariana Islands, Palau Island, Reunion, El Salvador, St. Lucia, US Virgin Islands, Albania, Estonia, Kiribati, Christmas Island.

Seasonality

In order to take into account the high influenza incidence during winter period and low incidence during summer season, we include seasonality in the infection transmission of the model. We follow the approach described in refs. [13, 14, 15] by using a transmission parameter β which varies in time and depends on the geographic zone. The world is divided into three different climate zones - Northern hemisphere, Tropical zone and Southern hemisphere - as outlined by the Tropic of Cancer and the Tropic of Capricorn (see Figure S1).

To each city, we assign a scaling factor for the transmission parameter which depends both

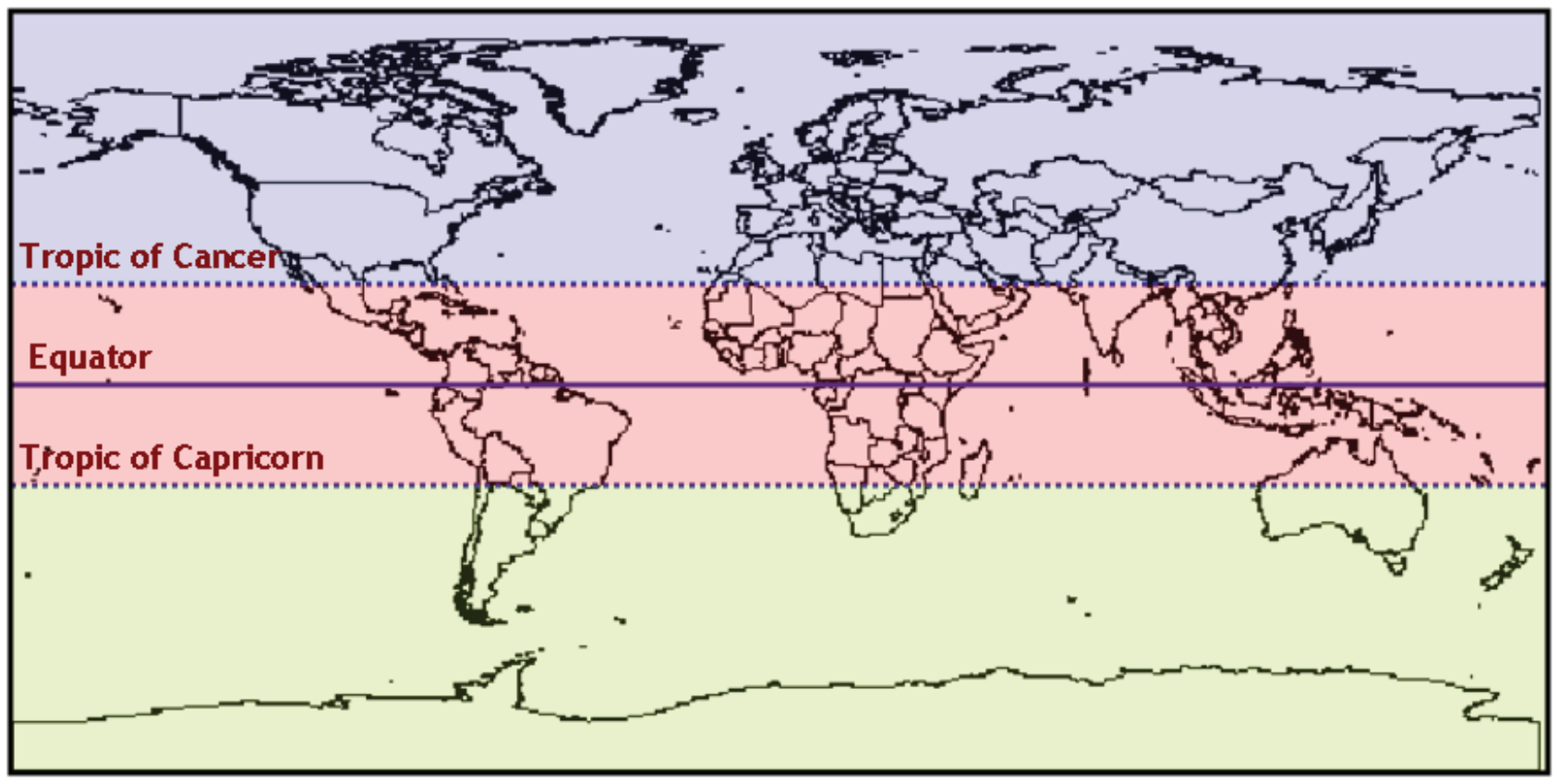

Figure S1: **Subdivision of the world into three climate zones – Northern hemisphere, Tropical zone, Southern hemisphere – as outlined by the Tropic of Cancer and the Tropic of Capricorn.**

on the time of the year and on the climate zone to which the city belongs to (see Table S2); its value models the seasonal variations of influenza. Following [15], we assume an enhancement of 10% of the transmission parameter β during the influenza season (November-February in the Northern hemisphere and May-August in the Southern hemisphere) and a reduction to as low as 10% during the non-influenza season (June-July in the Northern hemisphere and December-January in the Southern hemisphere). A linear interpolation between the two values is assumed to compute the scaling factor value for the remaining months. For all cities in the tropical climate zone, we assign a scaling factor equal to 1.

The 6 regions considered for the analysis of the results belong to the 3 climate zones introduced: North America and Western Europe to the Northern hemisphere, Central America and South East Asia to the Tropical climate zone, Lower South America and Oceania to the Southern hemisphere.

Month	Northern Hemisphere	Southern Hemisphere
January	1.1	0.10
February	1.1	0.35
March	0.85	0.6
April	0.6	0.85
May	0.35	1.1
June	0.10	1.1
July	0.10	1.1
August	0.35	1.1
September	0.6	0.85
October	0.85	0.6
November	1.1	0.35
December	1.1	0.10

Table S2: Seasonal scaling factors for the transmissibility β.

Epidemic evolution at the country and city level

The stochastic epidemic model introduced allows us to monitor the evolution of the pandemic in 3100 urban area belonging to 220 countries. While the manuscript shows agglomerated results for 6 different regions of the world, here we provide the corresponding time evolution of the pandemic at finer resolution scales - the country and city level.

Figures S2 and S3 characterize the time evolution of the disease spread in the baseline scenario by reporting the prevalence profiles of some countries and cities, respectively, chosen as illustrative examples. Results refer to a pandemic starting in Hanoi at the beginning of October. The virus propagates much faster for higher levels of infectiousness: while it would take almost one year and a half to spread globally around the world if $R_0 = 1.5$ (top panel of each figure), the larger value of infectiousness ($R_0 = 2.3$, bottom panels) would halve that time, with all countries and cities experiencing the first wave within 8-9 months.

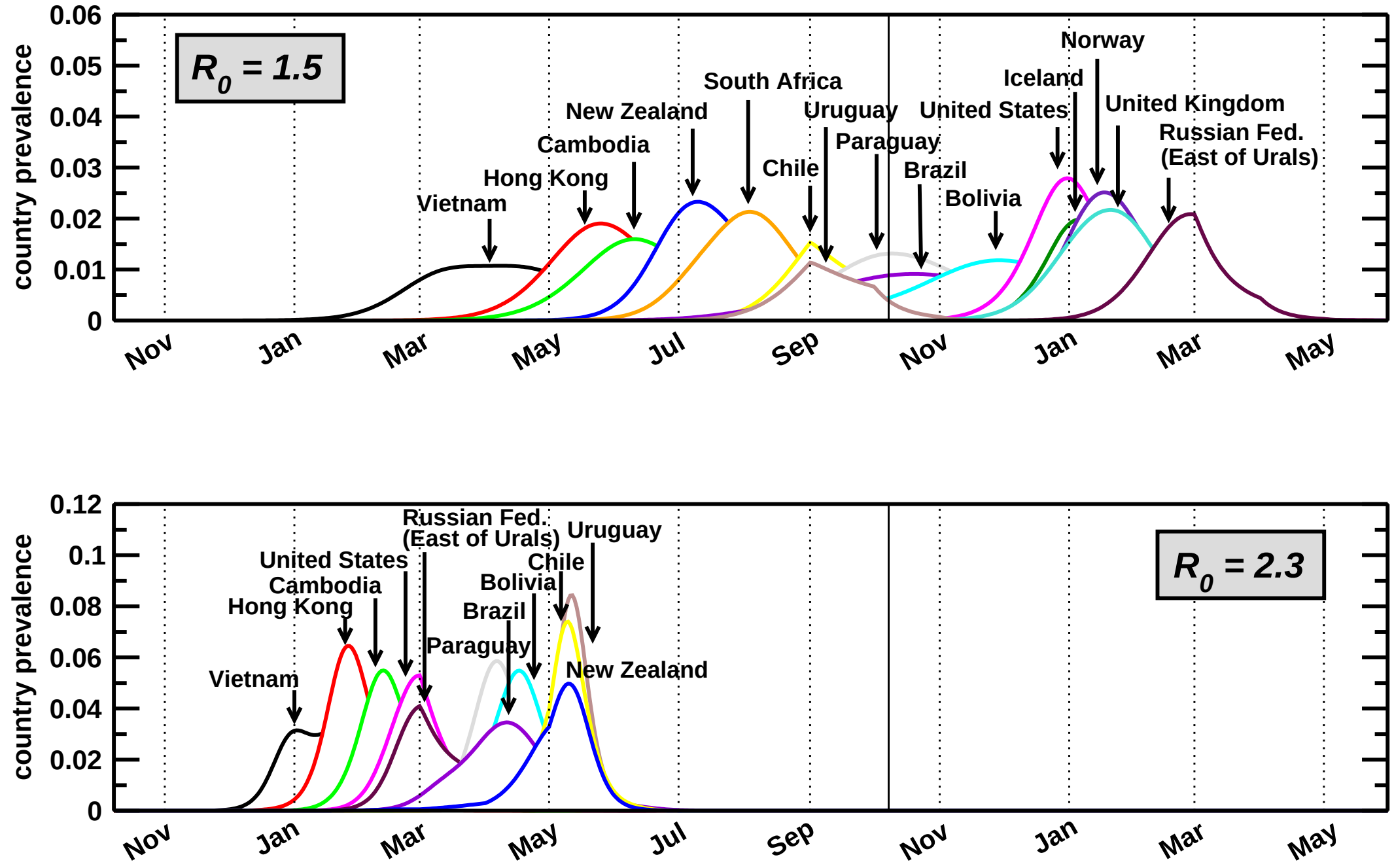

Figure S2: **Epidemic evolution in the baseline scenario at the country level.** Here we assume a pandemic starting in Hanoi in October, with $R_0 = 1.5$ (top panel) and $R_0 = 2.3$ (bottom panel). Countries are shown in both panels with the same color; not all countries reported for $R_0 = 1.5$ are shown in the bottom panel for sake of visualization.

Stochastic noise

In Figure S4 we report the same results shown in Figure 9 of the manuscript along with the stochastic noise associated to the average profiles. Here $R_0 = 1.9$ and the start of the pandemic is in Hanoi in October. Baseline and different intervention strategies are shown. Together with the average values - thick line corresponding to the baseline and symbols to different intervention scenarios - we also provide the 95% confidence interval, represented by the shaded areas. It is clear that when control strategies are in place the stochastic variability of a simulated influenza epidemic is relatively high, due to an effective reduction of the transmissibility.

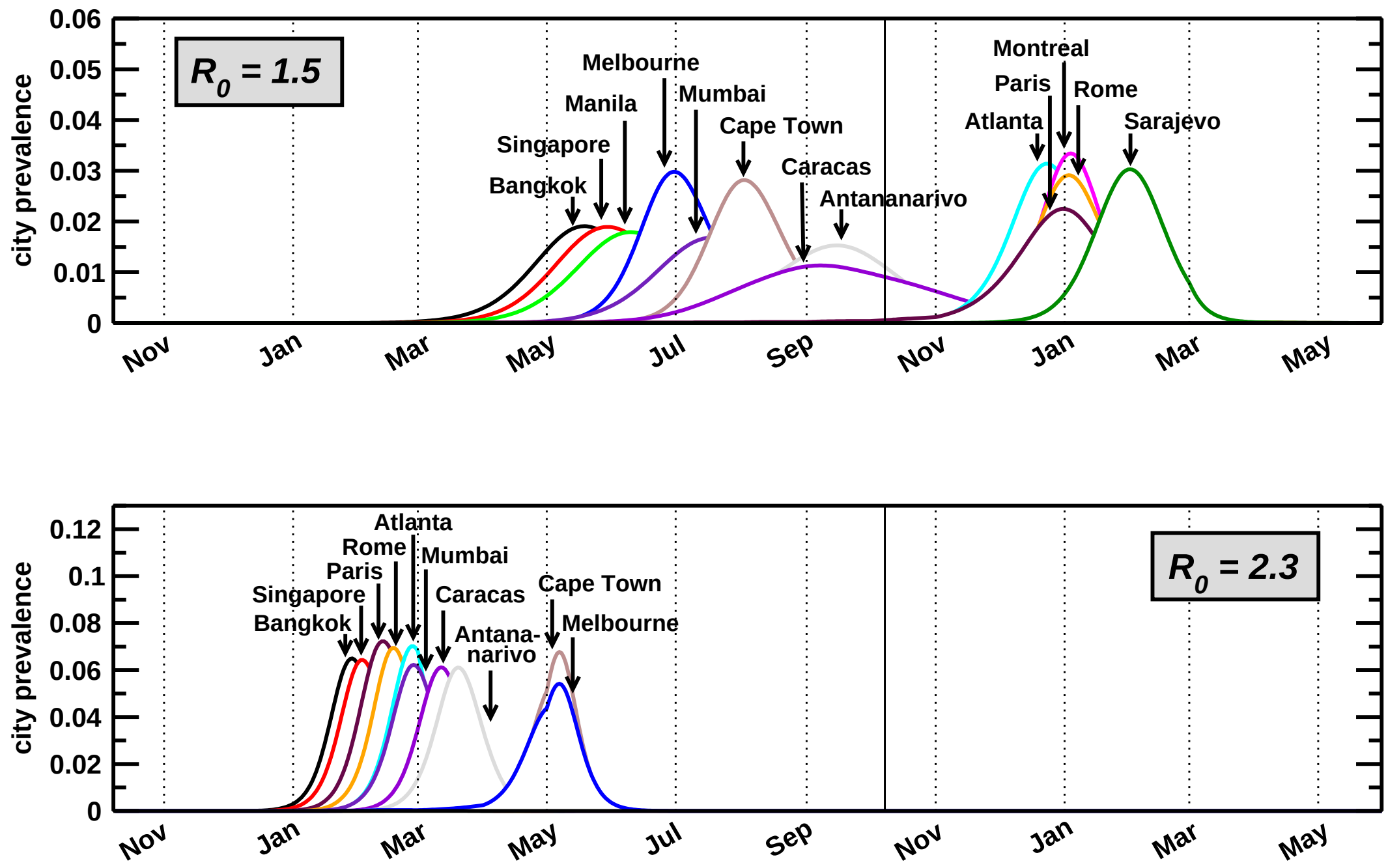

Figure S3: **Epidemic evolution in the baseline scenario at the city level.** Here we assume a pandemic starting in Hanoi at the beginning of October, with $R_0 = 1.5$ (top panel) and $R_0 = 2.3$ (bottom panel). Cities are shown in both panels with the same color; not all cities reported for $R_0 = 1.5$ are shown in the bottom panel for sake of visualization.

Sensitivity analysis

In the following we show the results obtained from the sensitivity analysis with respect to different factors characterizing the global spread of the pandemic as illustrated in the manuscript. More precisely, we study the effect of different temporal and geographical settings for the start of the pandemic in both the baseline and the intervention scenario; we also examine the role that the AV protocol assumed, the value of the reproductive ratio and the time delay of the intervention have on the outcoming results.

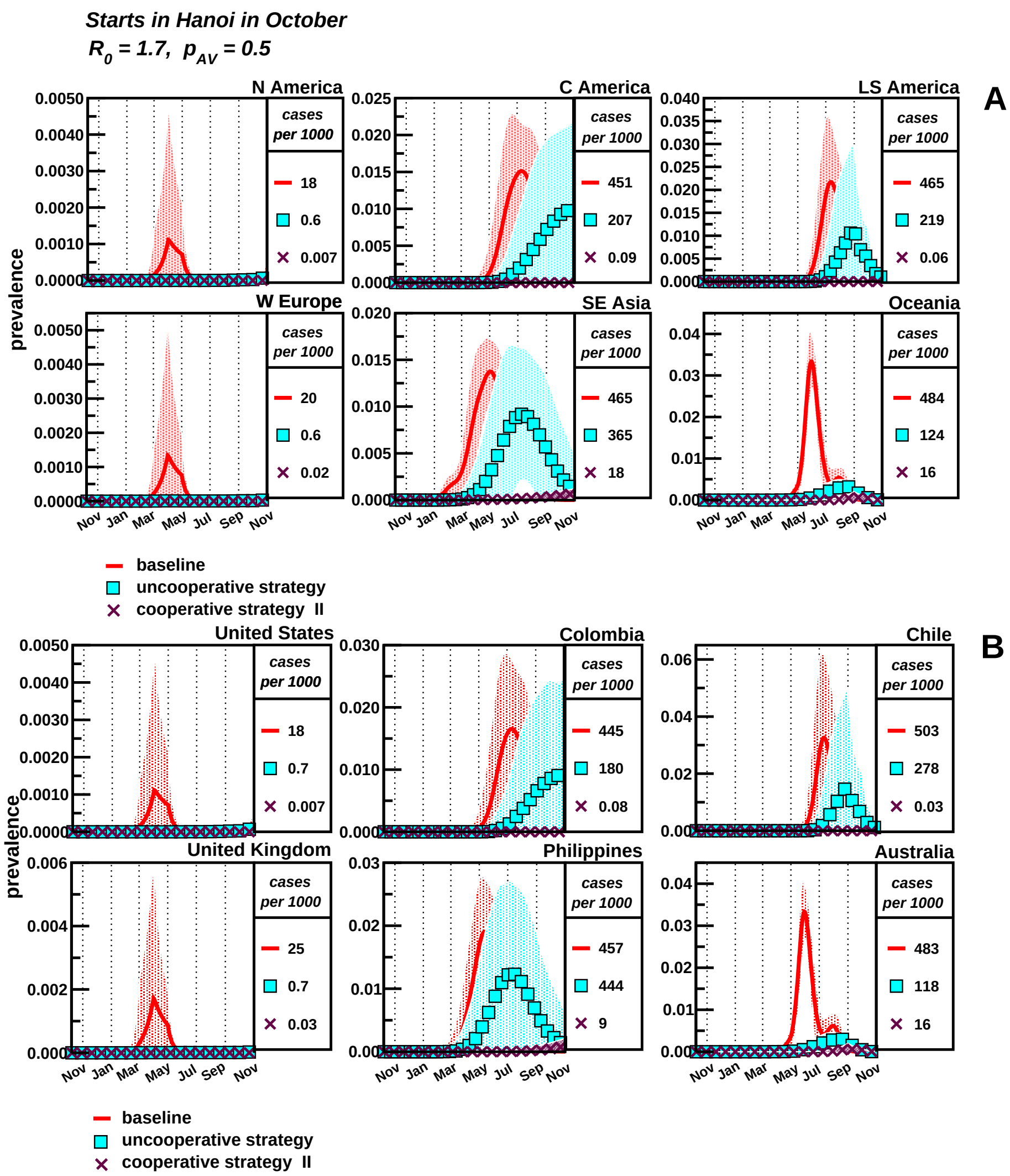

Figure S4: **Prevalence profiles for the baseline and the implemented intervention strategies with** $R_0 = 1.7$ **and** $p_{AV} = 0.5/day$**.** The average profiles are shown toghether with the 95% CI, represented by the shaded areas.

Baseline

Here we focus on the baseline scenario and present the effects of seasonality and of the transportation network in determining the global spread of the pandemic.

Initial seed and starting date

Figures S5 and S6 show the results for the baseline scenario for a pandemic starting in 2 different locations - Pleiku (Vietnam) and Chicago (US) - during 2 different seasons - October and April.

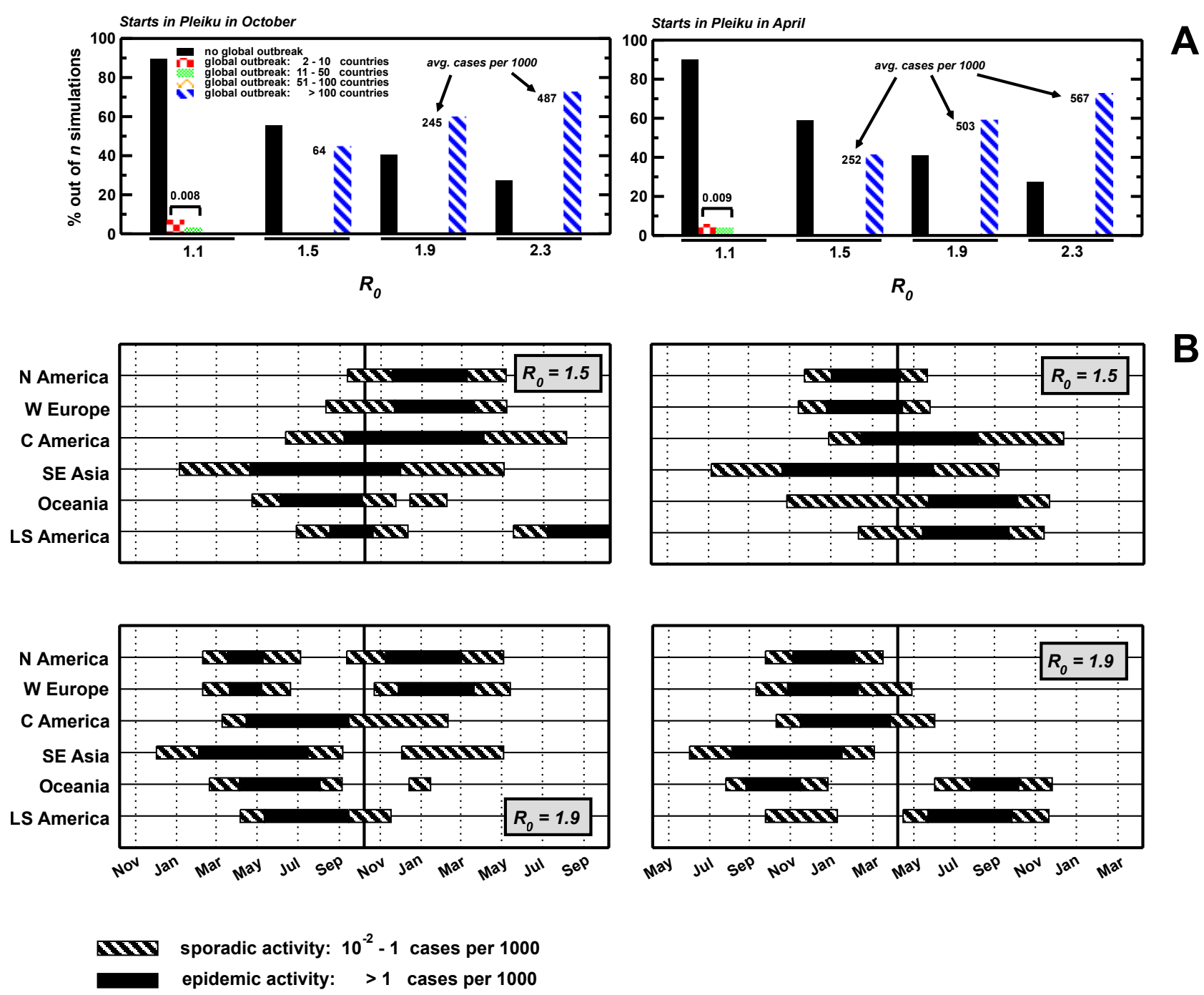

Figure S5: **Baseline scenario for a pandemic starting in Pleiku (Vietnam) in *October* or in *April***

We studied simulations in which the initially infected location is a small city in a rural area of Vietnam - Pleiku, i.e. the airport in Vietnam corresponding to the smallest urban area - since the pandemic might originate in a rural zone where population is living in close contact with poultry. Results of Fig. S5 show that no major differences are observed with respect to the case Hanoi is the starting location, except for a delay of the sporadic activity in the Northern hemisphere for $R_0 = 1.5$ and initial condition October. This is due to the lower connectivity of Pleiku with respect to Hanoi (smaller by a factor $\sim 1/10$) and in particular to the fact that

from Pleiku only national flights depart. For the virus to spread from Pleiku out of the country, two-leg flights are needed, while Hanoi is directly connected to several different airports of the world.

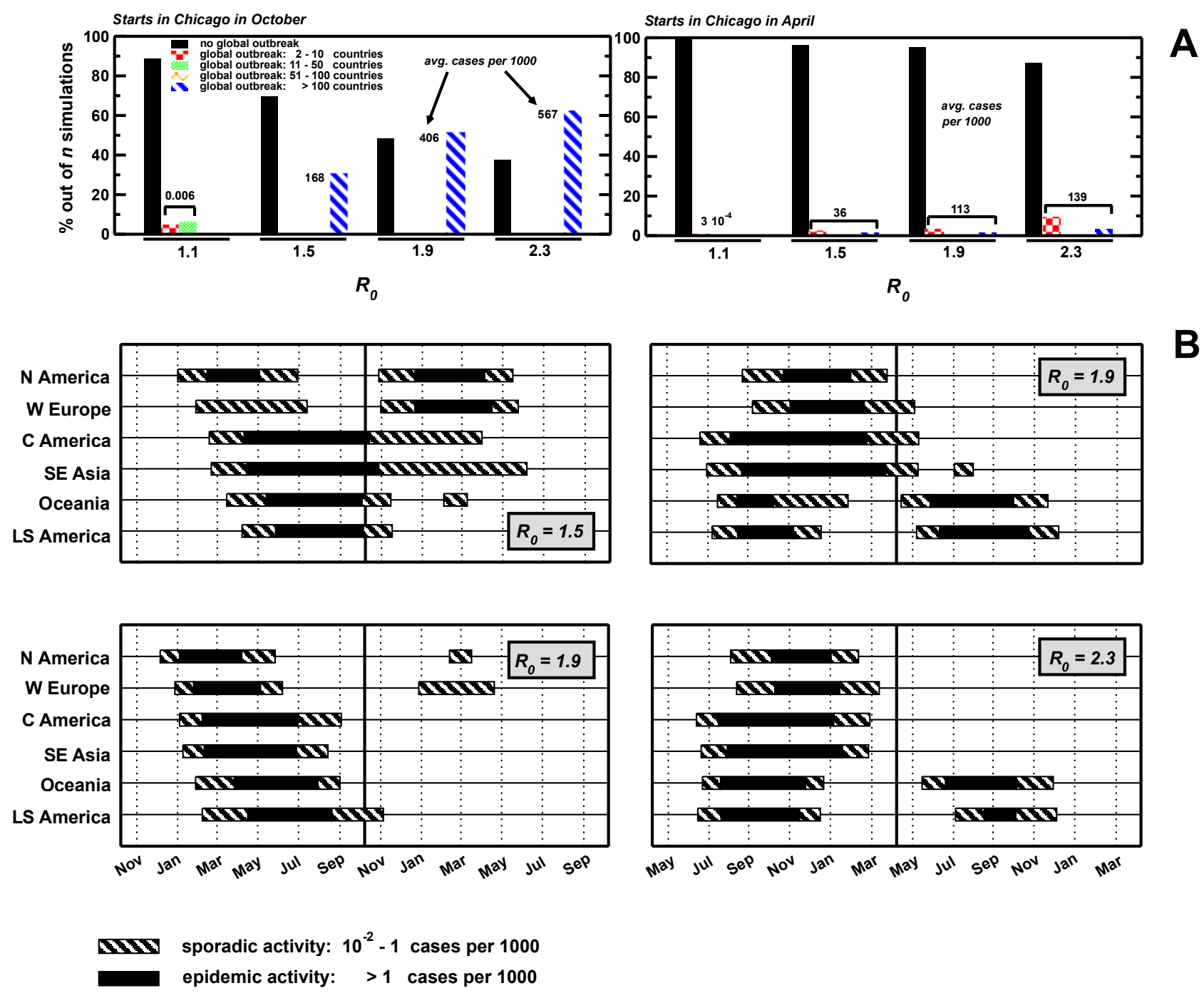

Figure S6: **Baseline scenario for a pandemic starting in the US (Chicago) in *October* or *April*.**

Fig. S6 shows the results of the simulations assuming Chicago as the starting city. The very low probability of having a global outbreak for a starting date in April underlines the effect of the seasonality, in agreement with what observed for Bucharest. However, differences from the Bucharest case (Figure 4 of the manuscript) point again to the role of the airline transportation network expressed in terms of the connectivity and traffic of the initially infected city. Chicago is ranked among the 10 airports in the United States with highest traffic; the number of passengers traveling on average through Chicago every day is more than one order of magnitude larger than the traffic going through Bucharest for the same period. This results on

average in a larger epidemic which produces higher numbers of cases in several regions which are better connected to Chicago than to Bucharest.

Intervention with AV therapeutic treatment: maximal coverage

This section reports the study of the sensitivity analysis of the time delays assumed in the model in case an intervention with maximal coverage is considered.

Time delay of the intervention

The model assumes that the pandemic alert is given after 20 cases are detected in at least one country. The intervention then starts with a characteristic delay in the seeded country - where cases have been detected - and in the other countries which are then reached by the virus. Here we investigate the effect of changes in the delays of intervention, considering delays of 1, 2 and 4 weeks both for the seeded country and for the seconday outbreaks. The AV protocol assumes a distribution rate $p_{AV} = 0.7/day$.

Analogously to the results obtained for the effect of the AV protocol, also in this case we observe an increase in the global attack rates after one year, with probability distributions for the number of infected countries almost unchanged. However, the increase observed is smaller than the one obtained by changing the distribution rate p_{AV}. Figures S7 and S8 show that a large delay in the intervention could be overcome with a prompt detection of symptomatic individuals and a high rate distribution of doses.

Intervention with AV therapeutic treatment: limited AV supplies

This section will analyze the effect of several different factors characterizing the intervention strategy through the use of limited AV supplies in the general scenario.

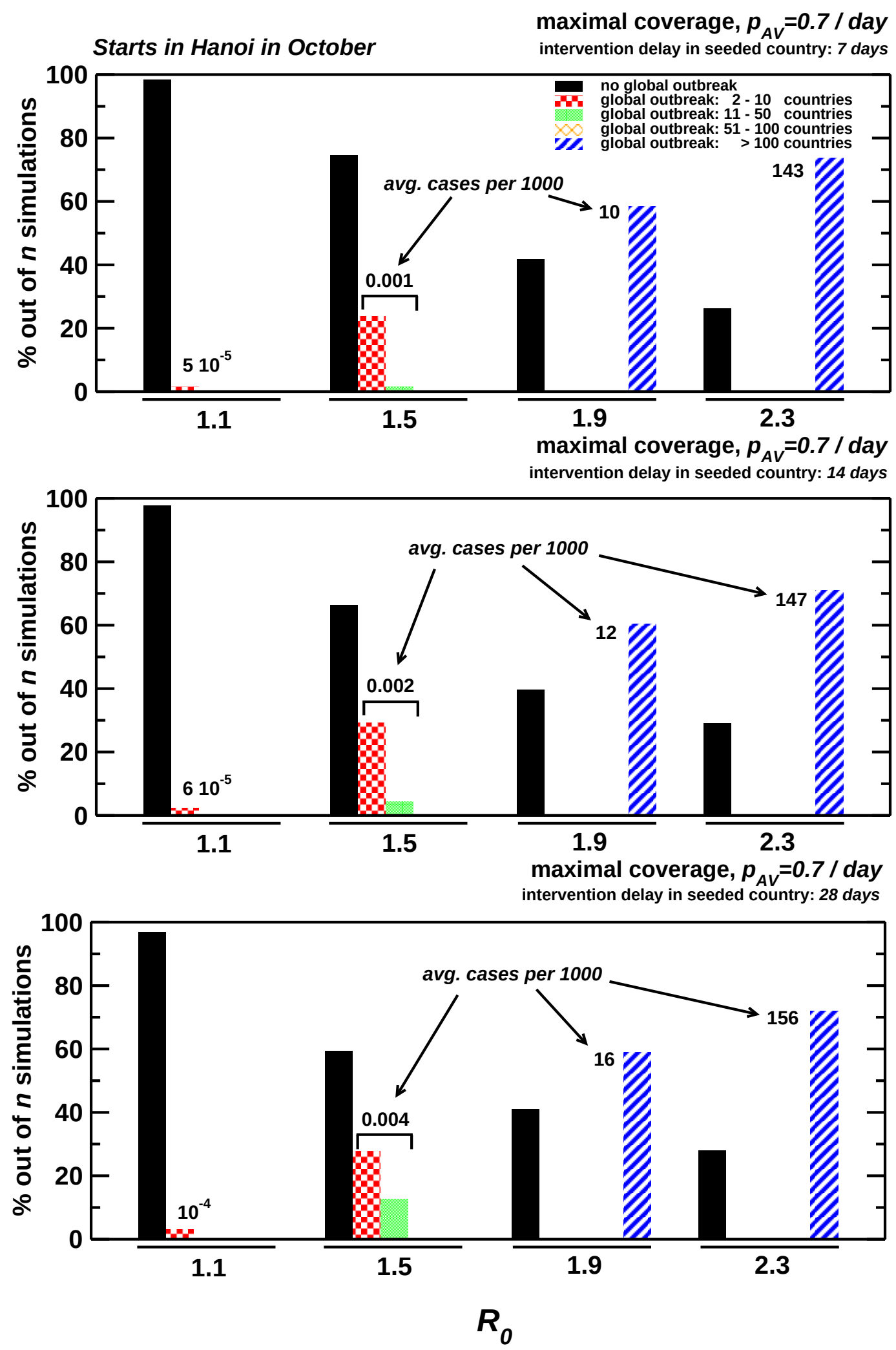

Figure S7: **Effect of different time delays of intervention in the initially infected country. The AV protocol assumed is $p_{AV} = 0.7/day$.**

Starting date

If the pandemic originates in Hanoi in April, no striking differences are observed with respect to the case discussed in the manuscript, where the starting date is October. As Figure S9 shows, we still observe a strong reduction of the number of cases per 1000 persons and a delay in the

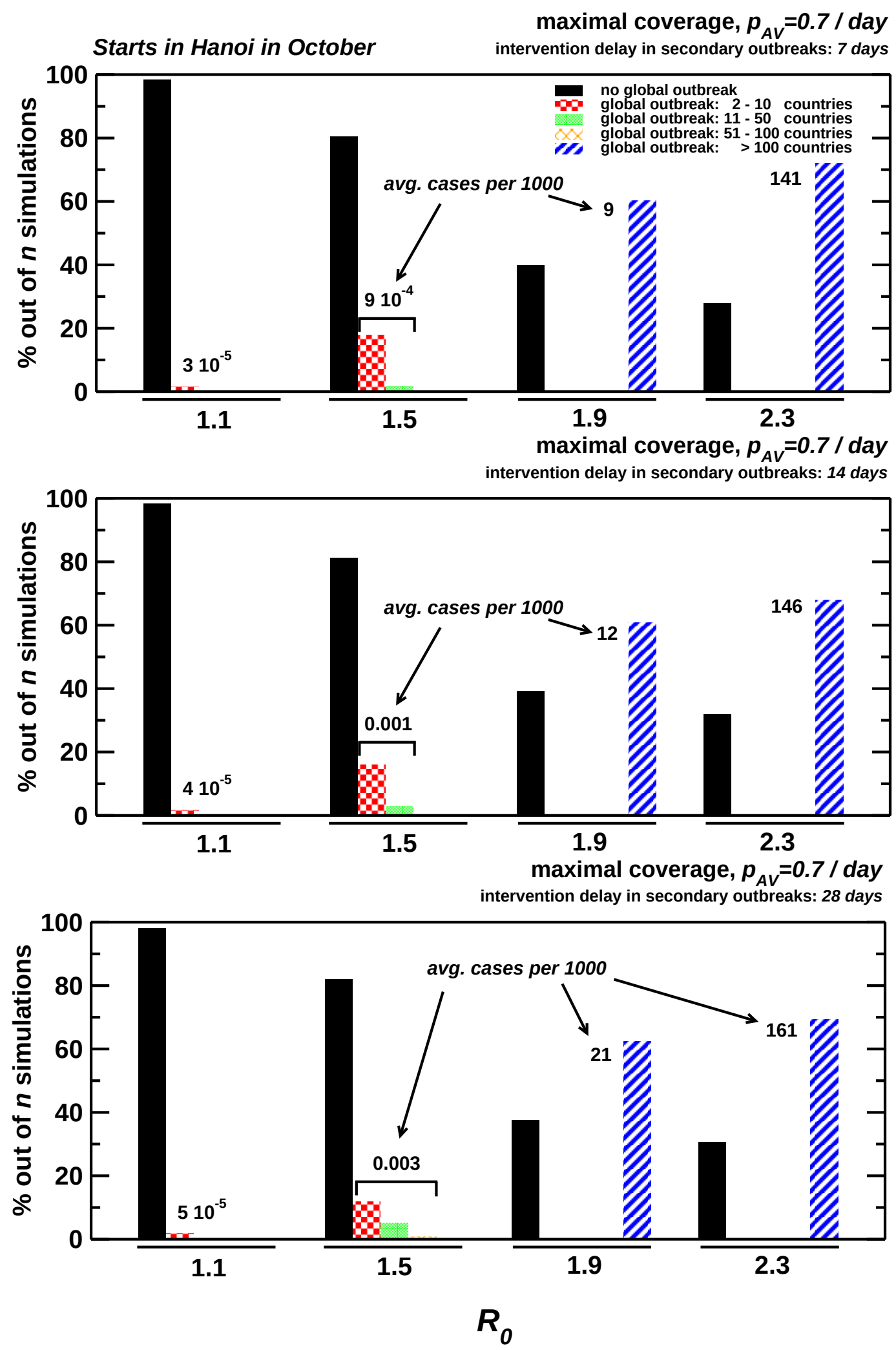

Figure S8: **Effect of different time delays of intervention in the countries experiencing secondary outbreaks. The AV protocol assumed is** $p_{AV} = 0.7/day$**.**

prevalence profile when the intervention is in place with respect to the baseline curve.

Initial seed

Results obtained for a pandemic seeded in Bucharest in October (Figure S10) are in agreement with the findings presented in the manuscript. In particular, it is possible to show that

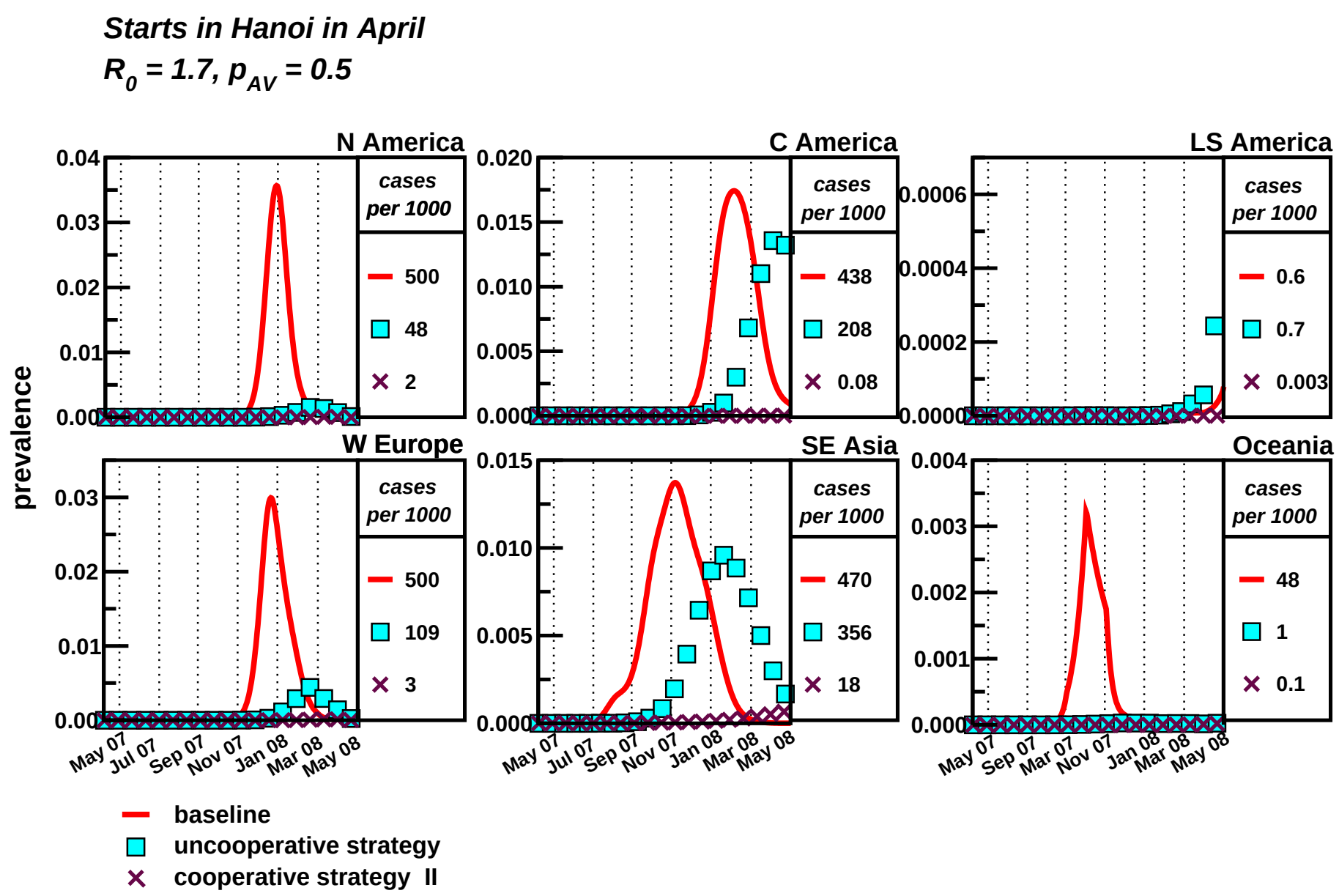

Figure S9: **Intervention scenario for a pandemic with $R_0 = 1.7$ starting in Hanoi in *April*. AV drugs are administered with rate $p_{AV} = 0.5/day$.**

cooperative strategies are extremely effective also in those regions where the application of an uncooperative strategy results in a delay of 2-4 weeks at most, with almost no reduction in the number of cases (see e.g. Central America, South East Asia and Lower South America). The attack rates are reduced by more than one order of magnitude also in the regions which provide part of their resources for global sharing.

Redistribution of shared AV resources

Here we analyze a different method of redistribution of the shared AV resources provided by the prepared countries. Instead of being used upon need, AV stockpiles are preemptively distributed to each of the unprepared country proportionally to its population.

Figure S11 shows that this solution is less effective with respect to the distribution of shared resources from a unique global stockpile for the country use when in need. Indeed, while some

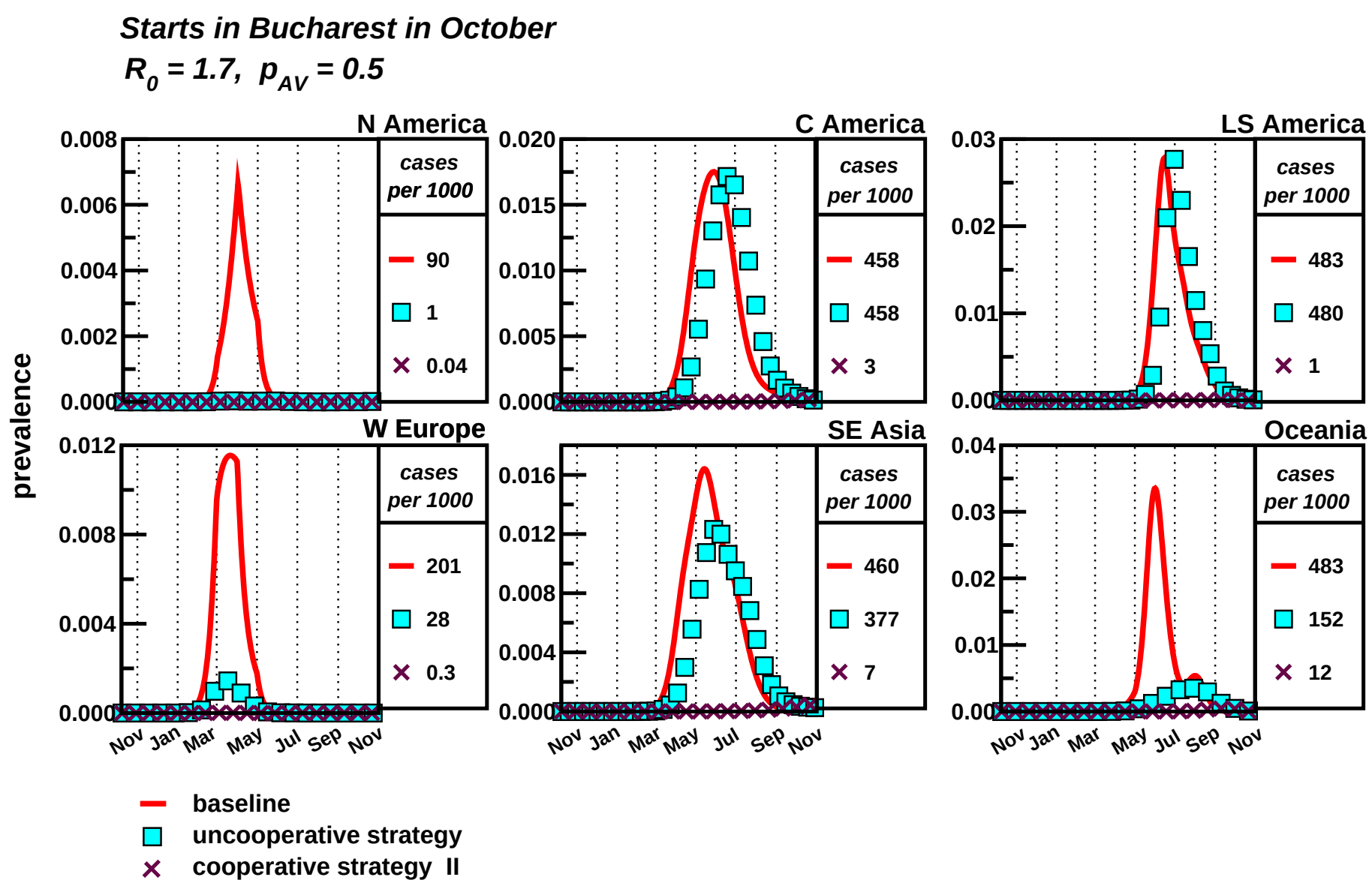

Figure S10: **Intervention scenario for a pandemic with $R_0 = 1.7$ starting in Bucharest in *October*. AV drugs are administered with rate $p_{AV} = 0.5/day$.**

of the more affected countries might run out of AV doses, some others might not use the whole amount received, at least during the first year. This creates a lack of resources in some regions (see e.g. SE Asia), which could be better optimized by a redistribution according to the actual need of each country.

Reduction of the infectious period during AV treatment

Results presented in the manuscript assume that the reduction of the average infectious period induced by the AV treatment is of 1 day. Here we study the impact of the efficacy of AV drugs as measured by its reduction of the infectious period. Following the details of the infectious period distribution as outlined in a previous section, we assume a reduction of $x = 0.5$ days.

While the global attack rates are larger than the ones obtained with $x = 1$ day, as expected, cooperative strategies are still more efficient than the uncooperative one in reducing the number

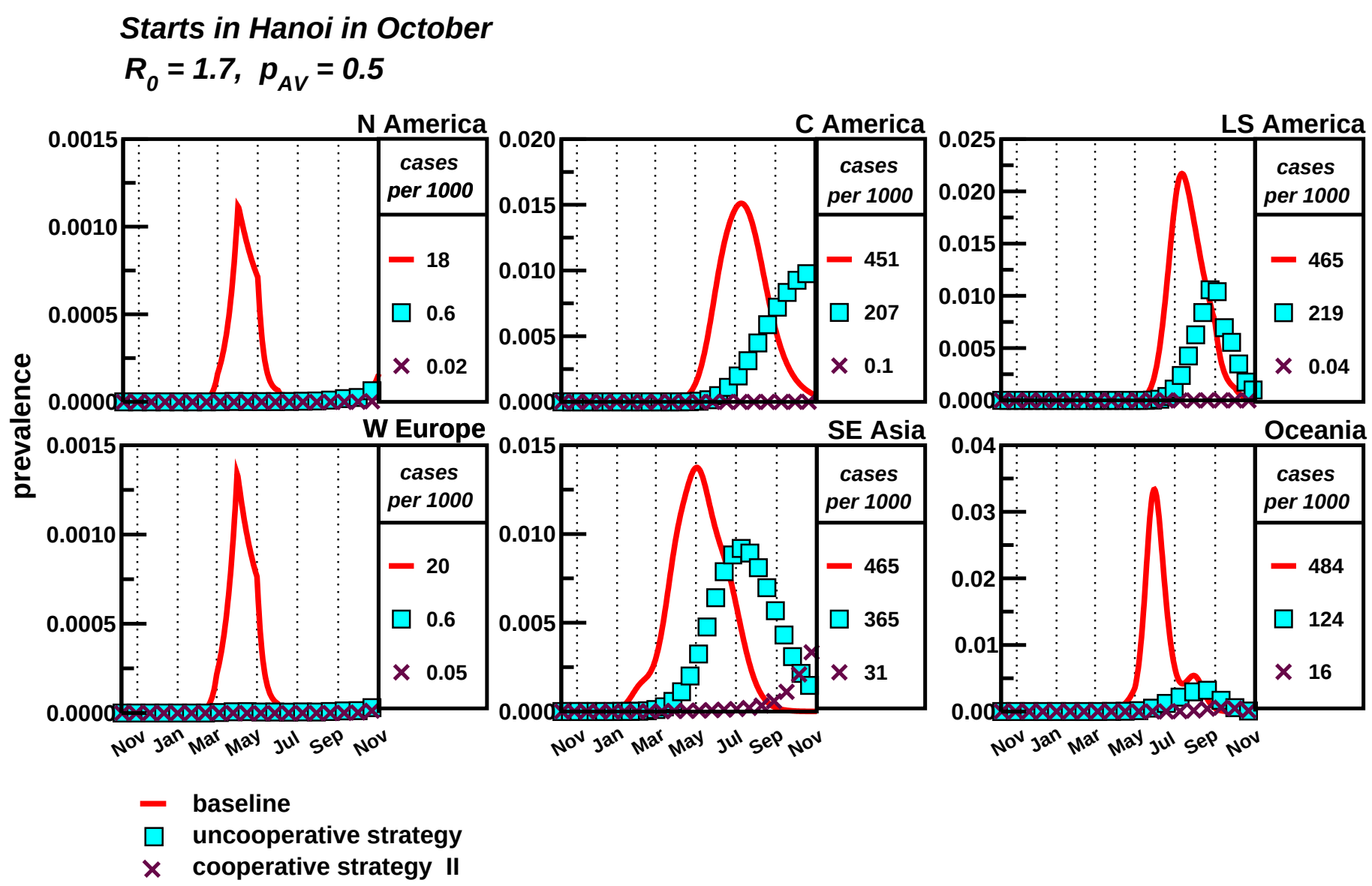

Figure S11: **Intervention scenario for a pandemic with $R_0 = 1.7$ starting in Hanoi in *October*. AV resources are assumed to be preemptively distributed in each country proportionally to the country population.**

of cases.

AV protocol and reproductive rate

Figures S13 and S14 display the effect of the AV protocol adopted on the containment of the disease, for different values of the reproductive rate. Results shown stress the crucial importance of the type of protocol to be adopted in order to better mitigate the disease impact. For $R_0 = 1.9$ the situation observed in the Northern hemisphere (North America and Western Europe) is almost unchanged, while other regions would experience the epidemic peak during the first year if $p_{AV} = 0.5/day$ instead of delaying it to the second year as shown for $p_{AV} = 0.7/day$. The global attack rates are therefore dramatically affected by the change of distribution rate. In addition, some of the prepeared countries, such as those in Oceania which are taking advantage of a large stockpile (10% of the population in the uncooperative strategy and 8% in the

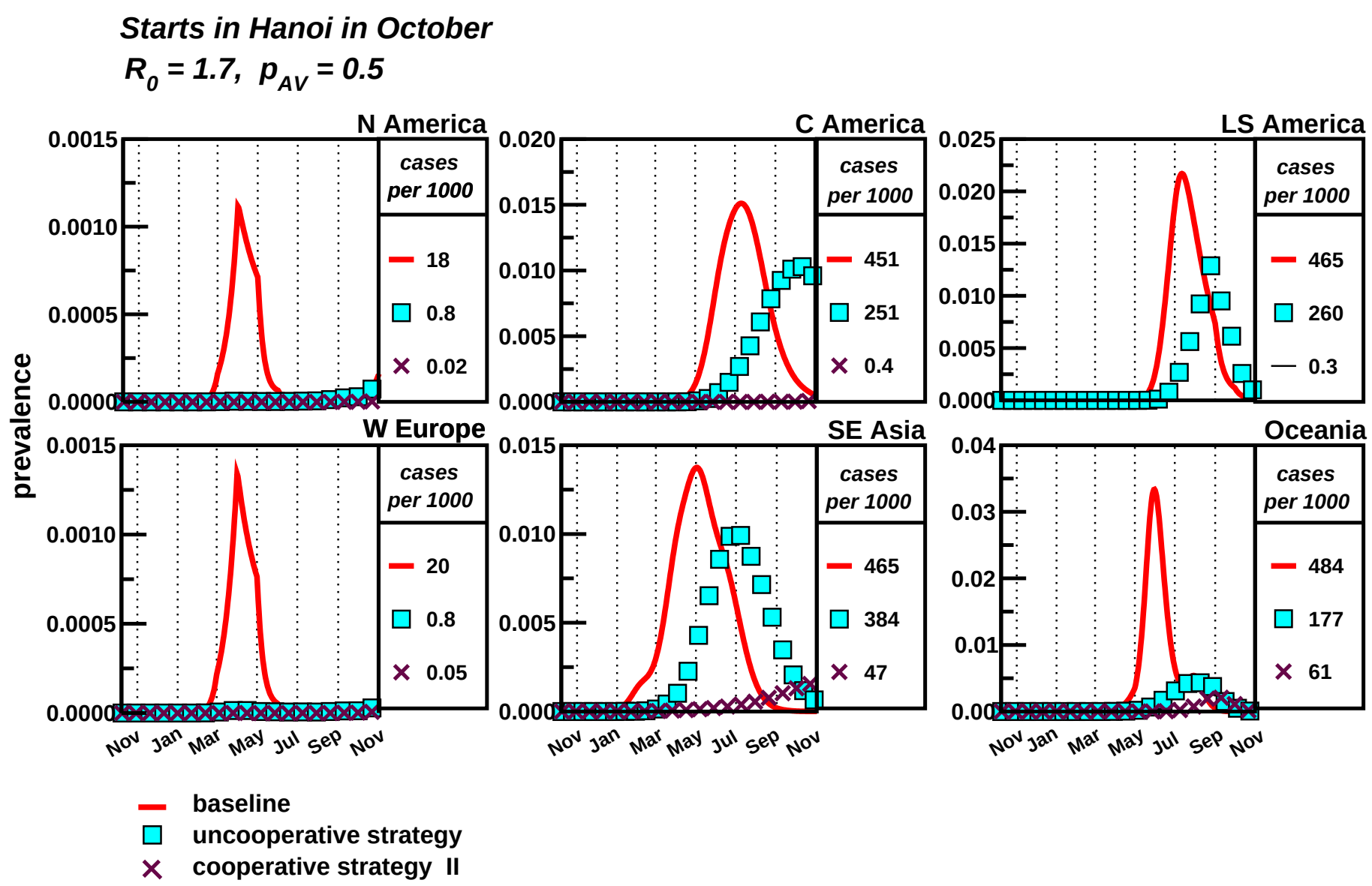

Figure S12: **Intervention scenario for a pandemic with $R_0 = 1.7$ starting in Hanoi in *October*. AV drugs are assumed to reduce the infectious period of 0.5 days.**

cooperative strategy II), are not able to consistently reduce the attack rate as in the case when $p_{AV} = 0.7/day$, due to the high traffic air connections with South East Asia which is experiencing a large outbreak. This clearly shows that, given the same amount of AV doses along with the same distribution of resources, it is preferable to treat the largest possible percentage of ill individuals in the unit time. Since we are dealing with finite and limited resources, one could actually expect that a better protocol would be to distribute a lower percentage of doses, in order to keep the available supplies for as long as possible. Results show that this is not the case. Indeed, if the intervention assumes $p_{AV} = 0.5/day$ instead of a higher rate value, an explosion of cases will occur in some regions so that the resources will not last longer and the observed attack rate will be higher. Finally, if the reproductive rate is higher, the most important advantage of the ccoperative strategies is in the delay of the epidemic peak. Indeed, in this case many regions would experience the peak within the first 12 months, so that the global attack rates are not strikingly reduced as observed for a lower R_0, but the vaccine development would

still benefit of an additional delay of 1-2 months with respect to the uncooperative strategy.

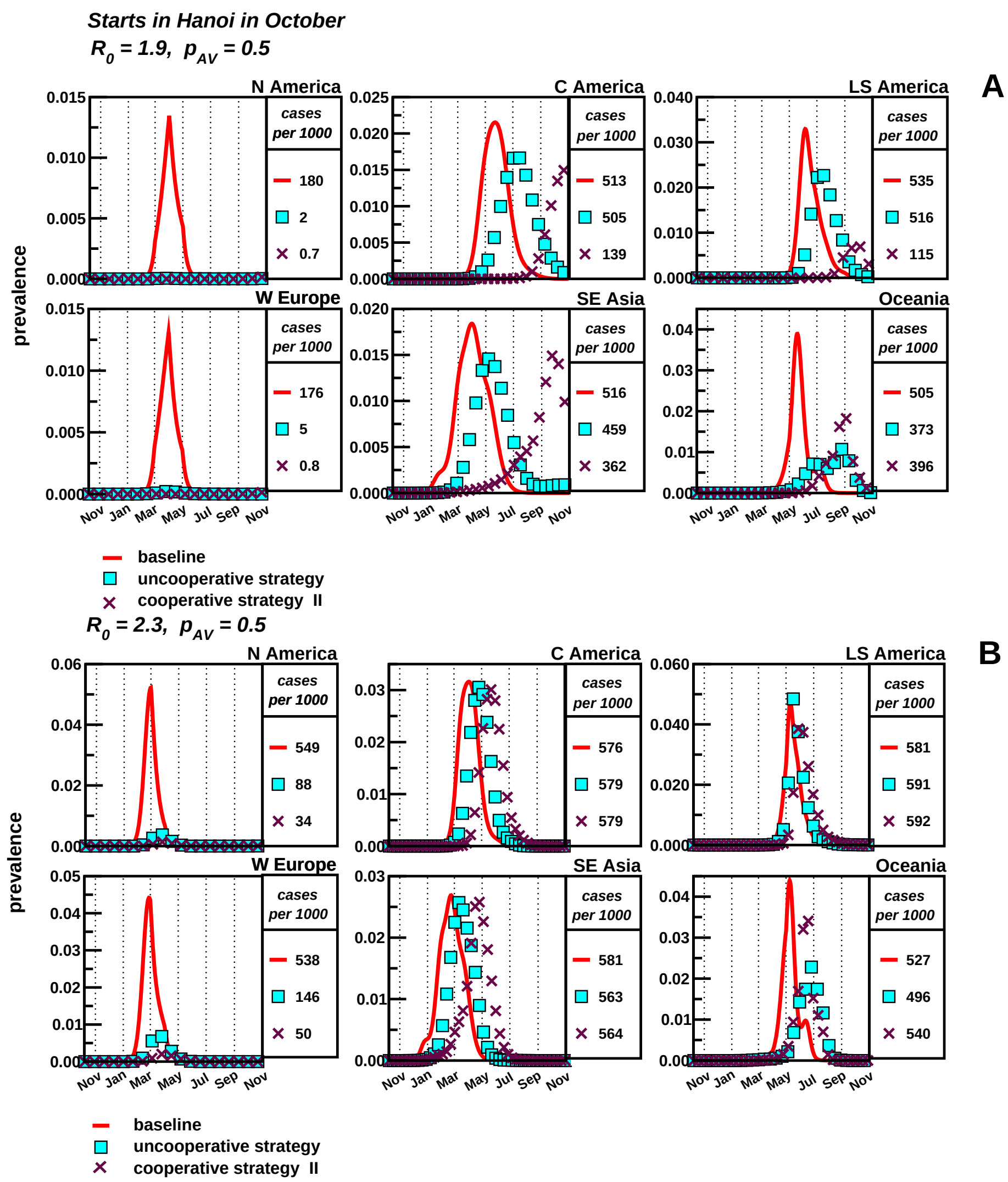

Figure S13: **Effect of different values of the reproductive ratio on the intervention strategies, when a distribution rate of $p_{AV} = 0.5/day$ is assumed.**

Time delay of the intervention

Figure S15 and S16 show the effect of the time delay in the application of the intervention strategy since the start of the pandemic. We performed numerical simulations assuming the

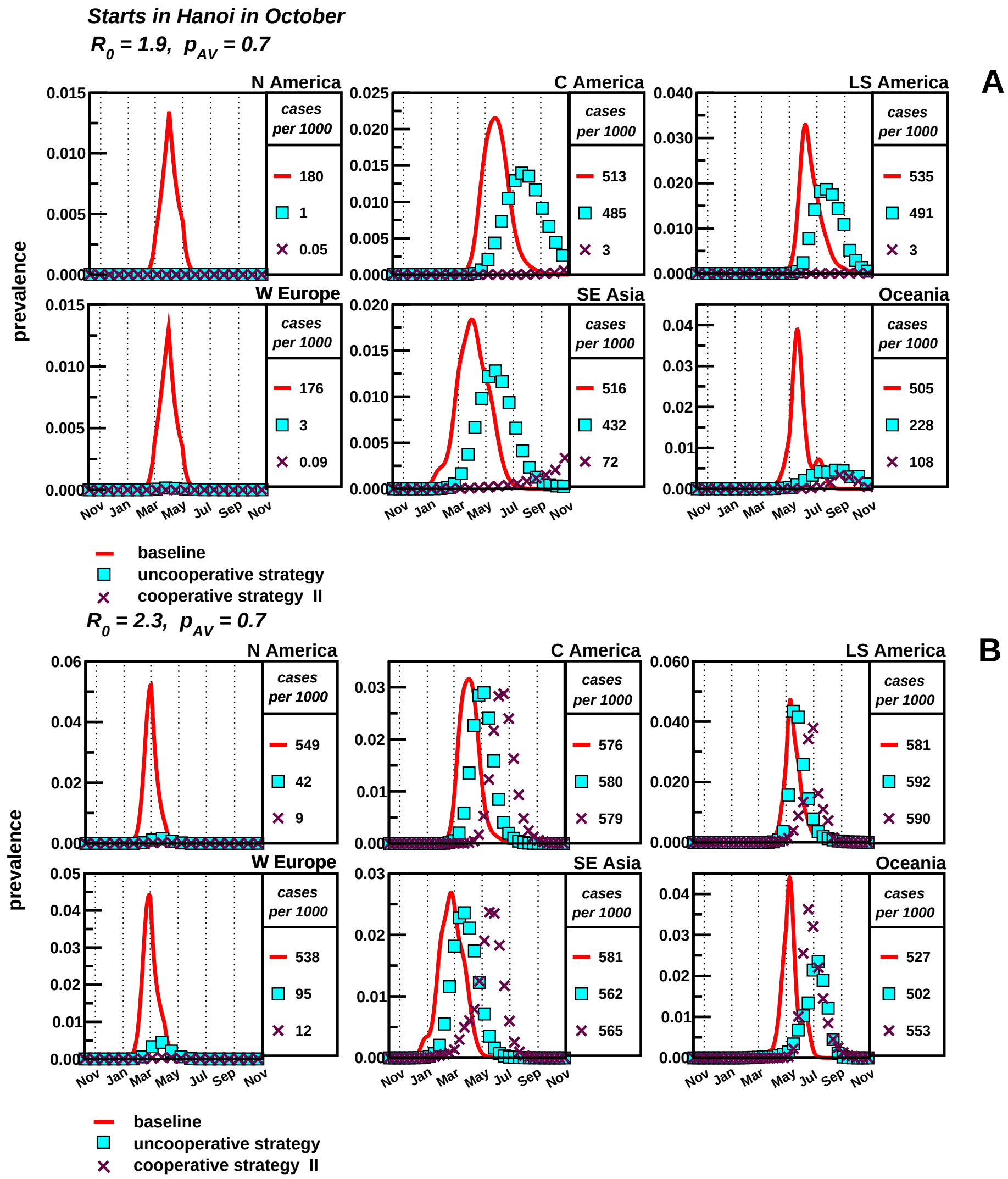

Figure S14: **Effect of different values of the reproductive ratio on the intervention strategies, when a distribution rate of $p_{AV} = 0.7/day$ is assumed.**

uncooperative strategy and the cooperative strategy II, with a protocol of $p_{AV} = 0.5/day$; the delays considered are 7, 14 and 28 days, corresponding to 1, 2 and 4 weeks respectively. Intervention delays both in the seeded country - after the detection of 20 infectious individuals - and in the secondary outbreaks are considered. As expected, larger values of the intervention delay correspond to larger and faster evolutions of the influenza pandemic throughout the world, with enhanced differences detectable in particular in the unprepared regions when the uncooperative

strategy is considered. Cooperative strategies are still more efficient with respect to the uncooperative one, providing an option that should be taken into account if the time delay for the onset of intervention is particularly large.

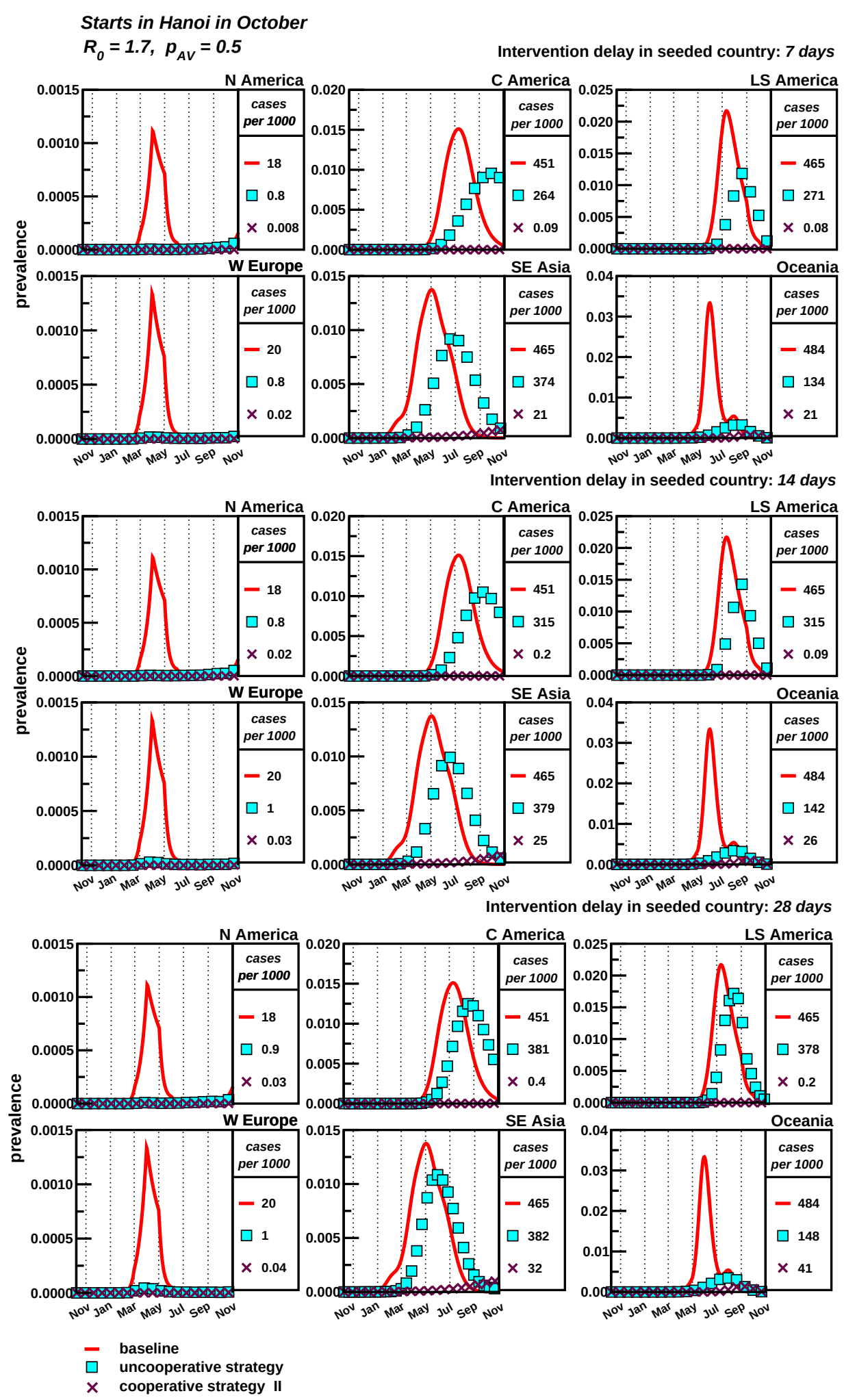

Figure S15: **Effect of different time delays of intervention in the initially infected country**. Here we consider a pandemic with $R_0 = 1.7$ starting in Hanoi in *October* with an assumed AV protocol of $p_{AV} = 0.5/day$. From top to bottom, results for a delay of 7, 14 and 28 days are shown.

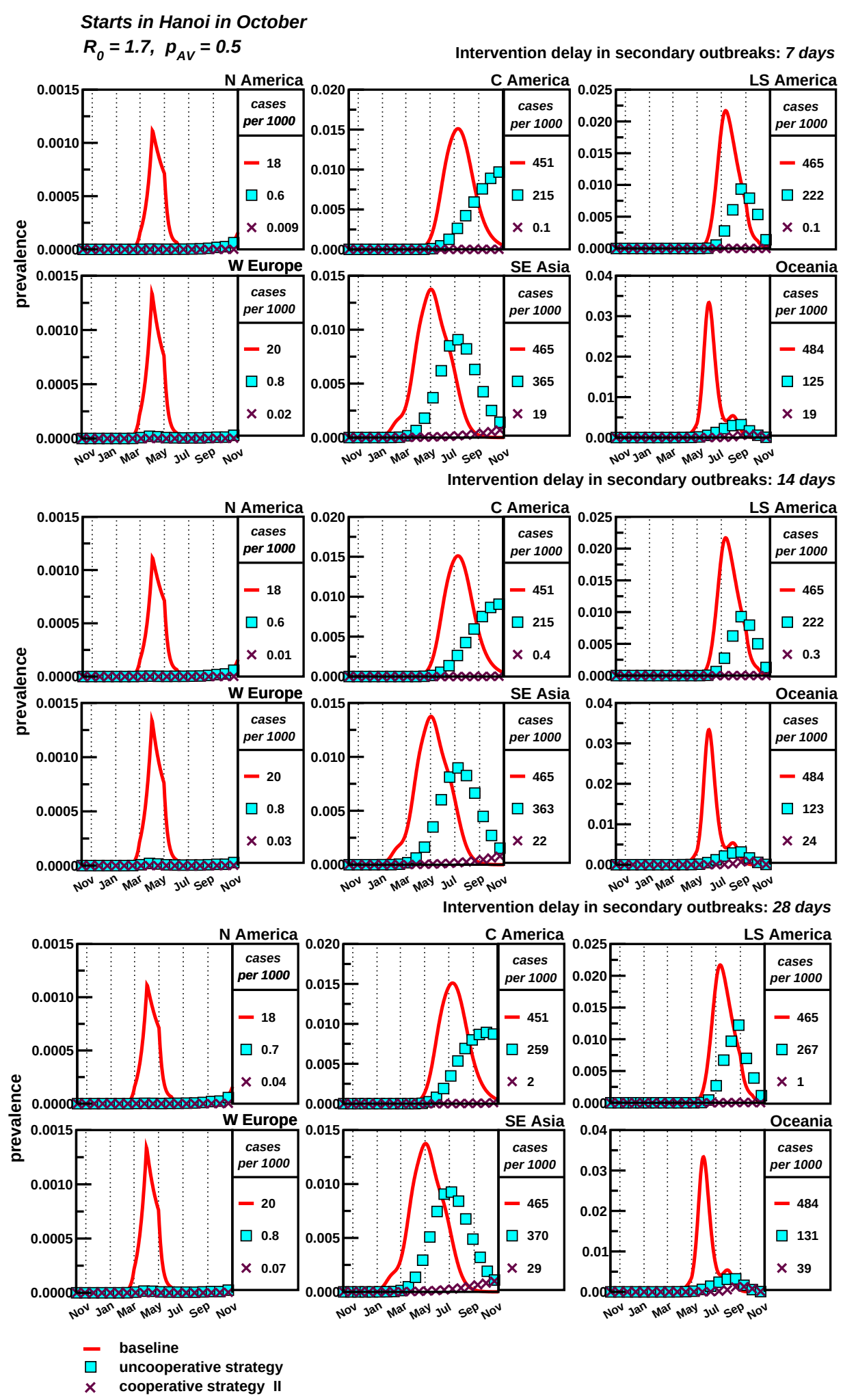

Figure S16: **Effect of different time delays of intervention in the countries experiencing secondary outbreaks.** Here we consider a pandemic with $R_0 = 1.7$ starting in Hanoi in *October* with an assumed AV protocol of $p_{AV} = 0.5/day$. From top to bottom, results for a delay of 7, 14 and 28 days are shown.

Travel limitations

While the enforcement of travel restrictions to and from infected areas is considered not applicable in most situations [11], it is reasonable to imagine that the air traffic volume in case of a pandemic will be reduced either due to the voluntary choice of individuals or for the applications of non-medical intervention measures. In Figure S17 we show what would happen in case

the number of passengers on each connection is reduced by 20% or 50%.

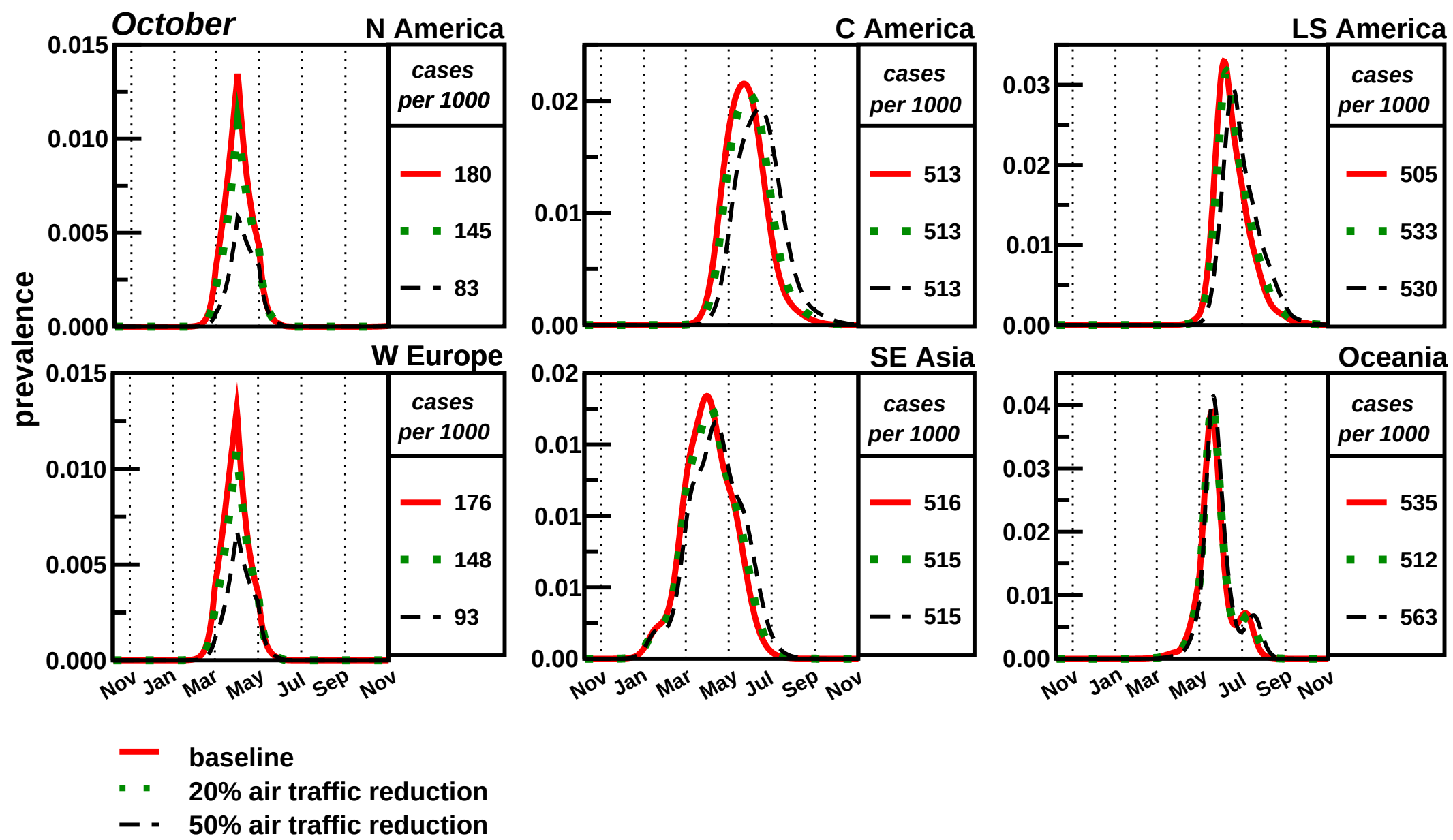

Figure S17: **Travel limitations as a non-medical intervention measure for a pandemic with** $R_0 = 1.9$ **starting in Hanoi in *October***

Although the traffic limitations imply a considerable reduction of air travel, the two intervention measures do not appear to be efficient neither in reducing the epidemic impact, nor in delaying the prevalence peak, in agreement with the results of refs. [20, 21]. The reduction in the air traffic is not able to contain the pandemic at the source. Indeed, as the outbreak occurs in the seeded country a larger number of cases is generated, so that the expected number of people traveling out of the country becomes considerably large though the probabilty of traveling has been drastically reduced. As soon as some infectious individuals travel from the source of the infection to another country, they will seed other regions with the infection, thus starting multiple outbreaks in different geographic zones (see the map reported in Figure 6 of the manuscript as an example). At that stage, the travel limitations are no longer able to considerably slow down the pandemic evolution.

Supporting Videos

Videos S1 and S2 represents the average simulated baseline scenarios corresponding to $R_0 = 1.7$ and $R_0 = 2.3$, respectively. The pandemic starts in Hanoi (Vietnam) in October. The color code represents the average number of cases per 1000 in each country on a logarithmic scale, from 10^{-2} (green) to 1000 (red). The videos show a time period of two years (video S1) and 14 months (video S2) since the start of the pandemic. Every snapshot represents the situation at the beginning of each month.

Video S3 reports the results obtained for the first 12 months spread of a pandemic with $R_0 = 1.7$ in case the uncooperative strategy is applied, where only few countries have AV stockpiles available for 10% of their population. Initial conditions are the same as in videos S1 and S2.

Video S4 represents the cooperative scenario II described in the main text, where 1/5 of prepared countries' stockpiles is globally redistributed. Initial conditions and infectiousness of the virus are the same as in the previous videos. The video shows the evolution during the first 12 months from the start of the pandemic.

References

[1] Colizza, V., Barrat, A., Barthélemy, M. & Vespignani, A. The role of the airline transportation network in the prediction and predictability of global epidemics. *Proc. Natl. Acad. Sci. USA* **103**, 2015-2020 (2006).

[2] International Air Transport Association (IATA), http://www.iata.org.

[3] Barrat, A., Barthélemy, M., Pastor-Satorras, R. & Vespignani, A. The architecture of complex weighted networks. *Proc. Natl. Acad. Sci. USA* **101**, 3747-3752 (2004).

[4] http://unstats.un.org/.

[5] http://www.census.gov/.

[6] Kilbourne, E.D. *The Influenza Viruses and Influenza*, Academic, New York (1975).

[7] Longini, I.M., Halloran, M.E., Nizam, A. & Yang, Y. Containing Pandemic Influenza with Antiviral Agents. *Am. J. Epidemiol.* **159**, 623 (2004).

[8] Longini, I.M., Nizam, A., Xu, S., Ungchusak, K., Hanshaoworakul, W., Cummings, D.A.T. & Halloran, M.E. Containing Pandemic Influenza at the Source. *Science* **309**, 1083 (2005).

[9] Ferguson, N.M., Cummings, D.A.T., Cauchemez, S., Fraser, C., Riley, S., Meeyai, A., Iamsirithaworn, S. & Burke, D.S. Strategies for containing an emerging influenza pandemic in Southeast Asia. *Nature* **437**, 209 (2005).

[10] Gani, R., Hughes, H., Fleming, D., Griffin, T., Medlock J. & Leach, S. Potential impact of antiviral drug use during influenza pandemic. *Emerg. Inf. Dis.* **11**, 1355 (2005).

[11] Avian Influenza: assessing the pandemic threat. World Health Organization (WHO). http://www.who.int/csr/disease/influenza/WHO_CDS_2005_29/en/.

[12] World Health Organization (WHO). http://www.who.int/.

[13] Rvachev, L.A. & Longini, I.M. A mathematical model for the global spread of influenza. *Mathematical Biosciences* **75**, 3 (1985).

[14] Grais, R.F., Hugh Ellis, J., & Glass, G.E. Assessing the impact of airline travel on the geographic spread of pandemic influenza. *European Journal of Epidemiology* **18**, 1065-1072 (2003).

[15] Grais, R.F., Hugh Ellis, J., Kress, A. & Glass, G.E. Modeling the spread of annual influenza epidemics in the US: the potential role of air travel. *Health Care Management Science*, **7**, 127-134 (2004).

[16] Gardiner, WC (2004) Handbook of Stochastic Methods for Physics, Chemistry and Natural Sciences, 3rd Ed. Springer, New York.

[17] Gillespie, DT (2000) The chemical Langevin equation. *J. Chem. Phys.* **113**, 297–306.

[18] Marro, J, Dickman, R (1998) Nonequilibrium Phase Transitions and Critical Phenomena. Cambridge University Press, Cambridge, UK.

[19] Dickman, R (1994) Numerical study of a field theory for directed percolation, *Physical Review E* **50**, 4404–4409.

[20] Flahault A, Vergu E, Coudeville L, Grais R (2006) Strategies for containing a global influenza pandemic. Vaccine (in press).

[21] Cooper BS, Pitman RJ, Edmund WJ, Gay NJ (2006) Delaying the international spread of pandemic influenza. *PLoS Medicine* **3** 845-855.

[22] Germann TC, Kadau K, Longini IM, Macken CA (2006) Mitigation strategies for pandemic influenza in the United States. *Proc. Natl. Acad. Sci. USA* **103**, 5935-5940.
